# Circulating IgG Levels in SARS-CoV-2 Convalescent Individuals in Cyprus

**DOI:** 10.3390/jcm10245882

**Published:** 2021-12-15

**Authors:** Ioannis Mamais, Apostolos Malatras, Gregory Papagregoriou, Natasa Giallourou, Andrea C. Kakouri, Peter Karayiannis, Maria Koliou, Eirini Christaki, Georgios K. Nikolopoulos, Constantinos Deltas

**Affiliations:** 1 Department of Health Sciences, School of Sciences, European University Cyprus, Nicosia 2404, Cyprus; i.mamais@euc.ac.cy; 2 Biobank.cy Center of Excellence in Biobanking and Biomedical Research, University of Cyprus, Nicosia 1678, Cyprus; malatras.apostolos@ucy.ac.cy (A.M.); papagregoriou.gregory@ucy.ac.cy (G.P.); giallourou.natasa@ucy.ac.cy (N.G.); kakouri.andrea@ucy.ac.cy (A.C.K.); 3 Department of Basic and Clinical Sciences, University of Nicosia Medical School, Nicosia 1700, Cyprus; karayiannis.p@unic.ac.cy; 4 Medical School, University of Cyprus, Nicosia 1678, Cyprus; koliou-mazeri.maria@ucy.ac.cy (M.K.); christaki.eirini@ucy.ac.cy (E.C.)

**Keywords:** SARS-CoV-2, antibodies, IgG, COVID-19, Cyprus

## Abstract

Long-term persistence and the heterogeneity of humoral response to SARS-CoV-2 have not yet been thoroughly investigated. The aim of this work is to study the production of circulating immunoglobulin class G (IgG) antibodies against SARS-CoV-2 in individuals with past infection in Cyprus. Individuals of the general population, with or without previous SARS-CoV-2 infection, were invited to visit the Biobank at the Center of Excellence in Biobanking and Biomedical Research of the University of Cyprus. Serum IgG antibodies were measured using the SARS-CoV-2 IgG and the SARS-CoV-2 IgG II Quant assays of Abbott Laboratories. Antibody responses to SARS-CoV-2 were also evaluated against participants’ demographic and clinical data. All statistical analyses were conducted in Stata 16. The median levels of receptor binding domain (RBD)-specific IgG in 969 unvaccinated individuals, who were reportedly infected between November 2020 and September 2021, were 432.1 arbitrary units (AI)/mL (interquartile range—IQR: 182.4–1147.3). Higher antibody levels were observed in older participants, males, and those who reportedly developed symptoms or were hospitalized. The RBD-specific IgG levels peaked at three months post symptom onset and subsequently decreased up to month six, with a slower decay thereafter. IgG response to the RBD of SARS-CoV-2 is bi-phasic with considerable titer variability. Levels of IgG are significantly associated with several parameters, including age, gender, and severity of symptoms.

## 1. Introduction

The coronavirus disease 2019 (COVID-19) pandemic, a major global health issue caused by the severe acute respiratory syndrome coronavirus 2 (SARS-CoV-2), has already resulted in nearly 5 million reported deaths worldwide (https://COVID19.who.int/; accessed on 17 October 2021). Nevertheless, within approximately 20 months from the detection of the first cases, the scientific progress has been remarkable, with advancements in multiple areas, including virology, immunology, diagnostics, surveillance, epidemiology, clinical management, and prevention culminating in the development of effective and safe vaccines [1,2,3,4,5,6,7,8,9,10,11]. Research has also shed light on the immune response to SARS-CoV-2, the understanding of which is vital for infection control. A basic component of the SARS-CoV-2-related immune response is that of antibodies targeting viral proteins, notably spike (S) and nucleocapsid (N). Accumulating evidence has shown that immunoglobulins (Ig) class M (IgM) and A (IgA) appear in circulation in most cases of infection, they are detected within the first two weeks from symptom onset, they reach their maximum quantities between the 15th and 30th day, and disappear at approximately 45–50 days post symptom onset [12,13]. The kinetics of circulating IgG are similar but with a peak titer between the 16th and 50th day from the day of symptom onset, and a decline thereafter [12,14,15]. Of interest, IgG seem to remain in the blood for several months and are still detected at least one year after infection [5,16,17,18]. Although our knowledge about the humoral response to SARS-CoV-2 infection has improved substantially, there are still unresolved questions including the durability of antibody response, the exact role of neutralizing antibodies, and especially the correlates of protection.

The Republic of Cyprus (government-controlled area), a small European island country of approximately 900,000 people in the Southeastern Mediterranean, has experienced multiple epidemic waves of SARS-CoV-2. The first wave in spring of 2020 was successfully contained by non-pharmaceutical interventions with limited burden on the healthcare system and a low number of fatalities [19,20]. Surges of cases occurred again in the late summer and early fall of 2020, followed by a big wave that started in November and peaked at the end of the year. New restriction measures contained the spread but not entirely. Viral transmission increased again in late February 2021, with a new peak in late April 2021. Due to the circulation of the highly transmissible Delta variant, the incidence of SARS-CoV-2 was very high in Cyprus in the summer of 2021, and although more than 60% of the total population is fully vaccinated (https://vaccinetracker.ecdc.europa.eu/public/extensions/COVID-19/vaccine-tracker.html#uptake-tab; accessed on 17 October 2021), there is still ongoing transmission in the early fall of 2021. By 15 October 2021, around 120,000 people have been diagnosed with SARS-CoV-2 infection, more than 5500 individuals have been hospitalized with 600 admissions at intensive care units (ICU), and 559 people have lost their lives due to COVID-19 (https://www.pio.gov.cy/coronavirus/uploads/SHORT%20ENG_Report%20COVID-19%20Cyprus%2014Oct_FINAL.pdf; accessed on 17 October 2021).

Although much is known about the basics of SARS-CoV-2 epidemiology in Cyprus, there are still unanswered questions concerning the immune response of the Cypriot population to SARS-CoV-2. Understanding the development and durability of humoral immune response among residents in Cyprus is an important scientific and public health task. The aim of this work is to describe the antibody response (N-IgG and receptor binding domain (RBD)-specific IgG) to natural SARS-CoV-2 infection among people living in Cyprus and determine parameters associated with that response.

## 2. Materials and Methods

### 2.1. Study Population

The study sample consisted of volunteers, with or without previous SARS-CoV-2 infection based on participants’ self-report, who visited the Biobank of the biobank.cy Center of Excellence in Biobanking and Biomedical Research of the University of Cyprus. All reportedly SARS-CoV-2 convalescent participants reported that their SARS-CoV-2 infection was confirmed either by rapid antigen testing or via reverse transcription PCR (RT-PCR). The study required that participants were free of symptoms at the time of enrolment to the study and excluded volunteers if they experienced symptoms less than 15 days prior to their visit. Written informed consent was obtained from all study participants in accordance with the Cyprus National Bioethics Committee approval (approval code: EEBK/EΠ/2020/19). Volunteers with contradiction to venipuncture were excluded from the study. Participants under 18 years of age could participate in the study following parental informed consent.

### 2.2. Data Collection

Demographic, socioeconomic, epidemiological information, as well as information on clinical symptoms and medical history, were collected from all participants through in-person interviews using a standardized questionnaire.

### 2.3. Specimen Collection, Storage, and Testing for Anti-SARS-CoV-2 IgG Antibodies

Peripheral blood was drawn from each volunteer. Plasma, serum, and genomic DNA samples were aliquoted and stored at −80 °C for future use. Biological samples and all participant data were de-identified and coded for protection of personal data. Participants’ sera were tested for antibodies using the SARS-CoV-2 IgG II Quant assay of Abbott Laboratories (quantitative method). This is a chemiluminescent microparticle immunoassay that can be used on ARCHITECT i or Alinity i Systems and detects IgG antibodies that target the RBD epitope of the spike S1 protein of SARS-CoV-2. The assay seropositivity cut-off is 50 arbitrary units (AU)/mL. The manufacturer reports a 99.6% specificity and a sensitivity that ranges from 51.7% in the first week after symptoms develop to 99.4% ≥15 days post symptom onset. An independent evaluation of the assay showed 100% specificity and an overall sensitivity of 75.4% that became 95.5% 16–20 days post symptom onset [21]. For about one-third of the participants and before the quantitative assay became commercially available, serum specimens were also tested for the presence of IgG antibodies against the nucleocapsid N protein of SARS-CoV-2 using the chemiluminescent microparticle immunoassay SARS-CoV-2 IgG of Abbott Laboratories (qualitative method) on ARCHITECT i and Alinity I Systems [22]. A signal/cut-off (S/C) index equal to or greater than 1.4 indicates seropositivity. The specificity reported by the manufacturer is 99.6–100%, while the sensitivity ranges from 25% in the first week after symptom onset to almost 100% two weeks after the development of symptoms [22]. An independent evaluation of the assay showed 100% specificity and an overall sensitivity of 84.6% that became 95.2% 12 days post symptom onset [22].

### 2.4. Statistical Analyses

Descriptive analyses included frequencies and percentages for categorical variables and medians and interquartile ranges (IQR) for continuous variables. Chi-squared or Fisher’s exact tests, Kruskal–Wallis or Mann–Whitney *U* tests, and the Spearman coefficient of correlation were used in univariable analyses. Multivariable log-linear and logistic regression models were used to evaluate the association between the levels or presence of antibodies and various factors or covariates including demographic characteristics, symptoms, and hospital admission. A two-sided *p*-value < 0.05 was considered statistically significant. All statistical analyses were performed using Stata 16 (StataCorp. 2019. Stata Statistical Software: Release 16. College Station, TX, USA: StataCorp LLC.)

## 3. Results

In total, 1898 volunteers were enrolled in the study (19 November 2020–24 September 2021) and were tested for SARS-CoV-2 IgG antibodies. Of these, 1112 individuals (58.6%) had reportedly been infected with SARS-CoV-2 in the past, while 786 individuals (41.4%) were not aware of a previous SARS-CoV-2 infection. The median age of the participants was 46 years old (IQR: 35–57), 1126 (59.3%) were males, and most of them (*n* = 1413, 74.5%) were residents of Nicosia, the largest district in the Republic of Cyprus (Table 1). Individuals who reported a history of vaccination (40% were reportedly partly vaccinated and 36% fully vaccinated against COVID-19) were excluded from the analyses. The final dataset included 1132 individuals, of whom 969 (85.6%) had a self-reported history of previous SARS-CoV-2 infection and 163 (14.4%) did not. 

### 3.1. Assays Performance

**Figure 1:**
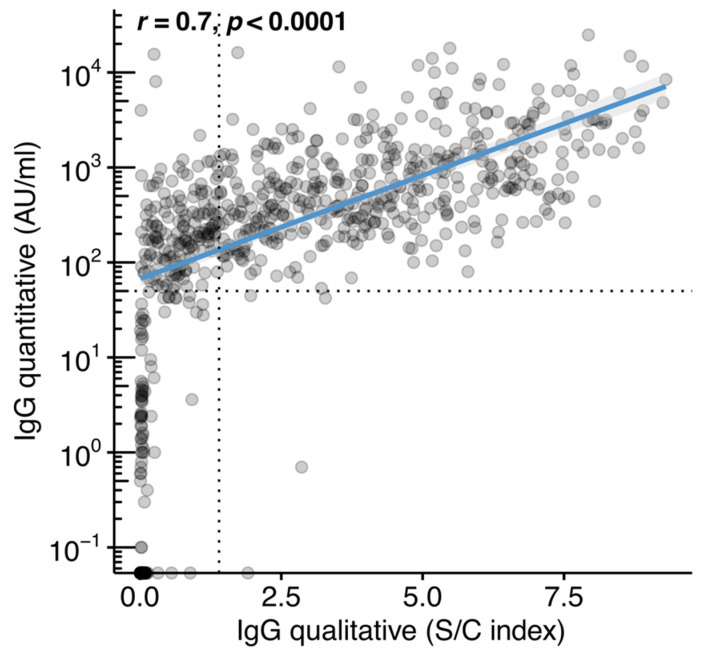
Correlation between the anti-SARS-CoV-2 immunoglobulin class G (IgG) quantitative and IgG qualitative assays. IgG (S/C) index plotted against IgG antibody levels in arbitrary units (AU)/mL. The analysis included 734 unvaccinated individuals with matched measurements for both assay types. Dotted lines represent the limits of detection for each assay and blue line represents linear regression fit. Spearman’s correlation test was used to calculate correlation coefficients (*r*) and *p*-value (*p*).

The study participants included in the final dataset were tested for IgG antibodies using the qualitative (*n* = 734, of whom 622 had a history of previous SARS-CoV-2 infection and 112 did not) and the quantitative method (*n* = 1132, of whom 969 had a history of previous SARS-CoV-2 infection and 163 did not) (Table 2). The qualitative method detected IgG seroconversion (≥1.4 S/C) in 431 of the participants (431/622 or 69.3%) who were reportedly infected by SARS-CoV-2. Most of the participants (107/112 or 95.5%) without a self-reported history of SARS-CoV-2 infection were negative for IgG antibodies against the N protein based on the qualitative method. When samples were tested by the quantitative method, the percentage of IgG seroconverters (≥50 AU/mL) among those who reported previous infection increased to 93.8% (909/969). There were 60 individuals (6.2%), 40 females (66.7%), who had reported a previous infection with SARS-CoV-2 but the result of the quantitative test was below the positivity cut-off (<50 AU/mL). These participants had a median age of 42 years (IQR: 33–51). The median time from reported symptom onset or from reported SARS-CoV-2 diagnosis to antibody testing was 77 days (IQR: 52–112) and 76 days (IQR: 54–109), respectively. Of these 60 participants, 21 (35%) had a history of one of the following groups of medical conditions: (a) cardiometabolic disease, (b) bone disease, (c) cancer, (d) kidney disease, and (e) respiratory disease. Of 163 individuals who were not aware of previous SARS-CoV-2 infection, the quantitative test was negative in 142 (87.1%). There was strong linear association (*r* = 0.7, *p* < 0.0001) between the S/C index of the qualitative assay and the IgG levels in AU/mL, as measured by the quantitative method (Figure 1).

### 3.2. IgG Antibody Level Durability

**Figure 2:**
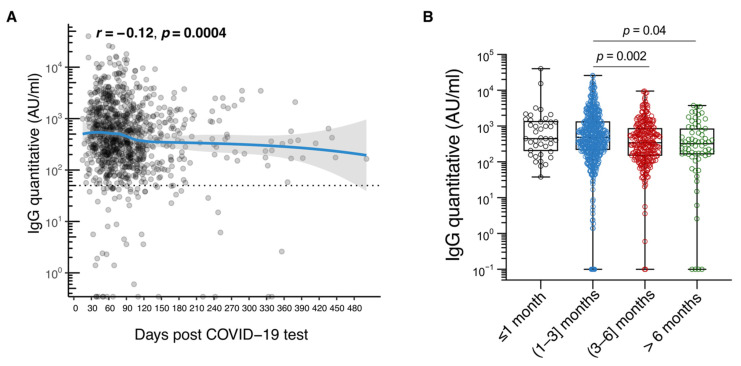
Immunoglobulin class G (IgG) antibody durability in the Cypriot population. (**A**). Plot of IgG levels in arbitrary units (AU)/mL against days since SARS-CoV-2 diagnosis; (**B**). Box plot of IgG levels in AU/mL ≤ 1 month, (1–3) months, (3–6) months, and >6 months post symptom onset. The analysis involved 969 unvaccinated people with a self-reported history of laboratory-confirmed SARS-CoV-2 infection.

There was also variation in median antibody levels over time (based on time either since symptom onset or since date of the first positive antigen or PCR test). The median RBD-specific IgG titer was 447.5 AU/mL (IQR: 207.6–1363.9) in the first month after symptoms appeared, increased to 495.0 (IRQ: 222.6–1407.0) between the first- and third-month post symptom onset, and then decreased to 354.0 (IQR: 153.6–894.4) from the third to the sixth month. After the sixth month, there was a reduction to a median value of 300.8 (IQR: 120.8–890.0) (Table 3, Figure 2A,B).

### 3.3. Correlates of IgG Antibody Response

Multivariable analyses using the results of the qualitative test showed that older age, male gender, development of symptoms, hospital admission, and the first three-month period since diagnosis or symptom onset were significantly associated with a higher likelihood of N-IgG seroconversion among the 622 unvaccinated individuals with a history of SARS-CoV-2 infection (Table 3). The analyses, based on the quantitative method run in 969 unvaccinated individuals with a history of SARS-CoV-2 infection, yielded similar results. The median titer of RBD-specific IgG antibodies in these participants with a self-reported history of SARS-CoV-2 infection was 432.1 AU/mL (IQR: 182.4–1147.3) (Table 2). The median RBD-specific IgG levels (AU/mL) were significantly higher in older individuals aged > 60 years (1413.7, IRQ: 567.4–3267.5) than in those aged 20–59 years (353.3, IRQ: 157.8–869.2), in males (529.8, IRQ: 206.8–1655.9) than in females (368.3, IRQ: 166.0–909.7), in those who developed symptoms (441.8, IQR: 187.1–1165.6), and especially fever and cough, than in those without symptoms (264.2, IQR: 46.9–636.5), and in hospitalized COVID-19 patients (2832.2, IQR: 1325.5–4165.4) compared to non-hospitalized patients (388.1, IQR: 171.8–989.0) (Table 3).

Four different multivariable linear regression models with logarithmic transformation of antibody levels (base 10) were also performed using the results of the quantitative method in the 969 unvaccinated participants with self-reported history of SARS-CoV-2 infection (Table 4). The overall model showed that older age (b = 0.020, *p* < 0.001), higher body mass index (BMI) (b = 0.015, *p*< 0.001), presence of symptoms (fever (b = 0.100, *p* < 0.001) and cough (b = 0.109, *p* < 0.001)), and hospital admission (b = 0.415, *p* < 0.001) were statistically significantly associated with a higher titer of RBD-specific IgG antibodies. Female gender (b = −0.102, *p* < 0.05), smoking (−0.201, *p* < 0.001), and time since self-reported date of laboratory diagnosis (b = −0.001, *p* < 0.001) were associated with significantly lower antibody levels. The R-squared of the overall model was 0.251.

## 4. Discussion

The immune response to SARS-CoV-2-infection has not yet been thoroughly understood. To enhance knowledge in the field, we collected biological samples and clinical information from 1898 volunteers through the Biobank of the biobank.cy Center of Excellence in Biobanking and Biomedical Research in Cyprus, and we evaluated the antibody levels of individuals to natural SARS-CoV-2 infection. The analyses focused on the RBD-specific IgG antibodies, which dominate immune responses [23] and are considered the most appropriate indicators of past infection with SARS-CoV-2, especially for population studies [24]. The analyses showed that RBD-specific IgG among previously infected individuals changed over time from the date of diagnosis and/or symptom onset. Specifically, antibody levels increased during the first three months and then decreased until month six, with a much slower rate of reduction thereafter. Furthermore, differences in the antibody levels across different population sub-groups were recorded, suggesting that the heterogeneity of immune responses including that of circulating antibodies is a salient characteristic of SARS-CoV-2 infection. In particular, our study showed that older individuals (>60 years), males, and those who developed symptoms or were hospitalized, were more likely to achieve higher titers of RBD-specific IgG or have IgG against the N protein.

Our results show that the concentration of circulating IgG antibodies decreases with time, but they remain detectable for more than six months after symptom onset or diagnosis, which corroborates the findings of other research groups. The initial decrease in the antibody levels, followed by a more gradual decline, is consistent with the bi-phasic nature of antibody kinetics, which describes the transition to the secretion of antibodies from virus-specific long-lived plasma cells in the bone marrow, rather than from short-lived plasmablasts, which are produced later in the immune response to SARS-CoV-2 infection [16,25,26]. Previous studies have examined the presence of S-specific IgG antibodies and have reported that antibody levels decrease during the first four to six months post infection but remain in circulation for up to 13 months, while N-specific IgG levels seem to decline faster, but with seroconversion still detected 13 months post infection [16,27]. In addition, IgG-secreting S-specific plasma cells were also detected in bone marrow aspirates in 15 out of 19 convalescent individuals more than seven months after infection, suggesting a long-lived humoral immune memory following a mild SARS-CoV-2 infection [16]. Similarly, RBD-specific and neutralizing antibodies were detectable in the circulation for up to a year post infection [5,27]. In terms of the circulating immune memory, which is important for protection from severe disease or death, more memory S-specific B cells were observed at six months compared to the first month after symptom development, with IgG being the dominant isotype. Virus-specific memory CD4 T cells, a feature of cellular immunity, were also detected in >90% of COVID-19 cases between six and eight months after infection [28]. Although we did not measure neutralizing antibodies, whose high titers can help humans accomplish sterilizing immunity, existing research suggests that they show high correlation with RBD-specific IgG antibodies, and we would therefore expect a similar trend in the Cypriot population [29,30,31,32].

Disease severity, including hospitalization, was associated with higher antibody production elicited by SARS-CoV-2. Our results are in line with previous studies that describe higher levels of total IgG, RBD-specific IgG, and S-specific IgG antibodies in hospitalized patients with severe COVID-19 compared to patients with mild/moderate disease and/or patients who were not hospitalized [23,33,34,35]. Interestingly, in one of these studies, the antibody responses of those with severe disease occurred on average one week later than in those with mild/moderate disease. This delay in antibody production during the early days of infection might explain the disease progression of severe COVID-19 cases. Furthermore, some of these studies showed that the median level of S-specific, RBD-specific IgG and neutralizing antibodies was higher in patients older than 60–65 years [33,34].

Comparison of the RBD-specific antibody levels between males and females showed that circulating IgG levels are higher in males. Similarly, previous studies showed increased titers of S-specific, RBD-specific, and N-specific antibodies in males, including analyses that were restricted to cases who did not require hospitalization [28]. Contrary to our findings, there are studies that detected higher levels of antibodies in females [12,36]. Specifically, one study in China enrolled 331 hospitalized individuals with confirmed SARS-CoV-2 infection and reported that the circulating IgG levels were higher among females with severe disease than among males of the same condition, while women were also more likely to present with elevated IgG antibody levels in the early stages (two to four weeks) of COVID-19 [36]. The latter finding could explain the increased likelihood of hospitalization, ICU admission, and death of males with COVID-19, although the cellular immunity should also be evaluated. The differences in IgG production between males and females may be explained in part by several factors, including differences in disease severity, sample size, detection methods, and other host factors [37]. Furthermore, differences in the immune response to infections, vaccinations, as well as in association with autoimmune diseases, between males and females have previously been reported and have been attributed to factors such as sex hormones, X-chromosomal and environmental factors [38].

Although gender, age, and disease severity contribute to the heterogeneity of immune response reported in our study, the source of much of the overall heterogeneity remains unknown and is worthy of further examination. Our overall multivariable model in Table 4 explained only a small portion of the variability in RBD-specific IgG levels, suggesting that other factors including genetics, antigenic load, immune status, comorbidities, and pre-existing immunity are likely important determinants too [39]. For instance, seroprevalence was reduced in people with inflammatory bowel disease (IBD) treated with infliximab [40] and IgG antibodies against the N protein were not detected in IBD patients four months after the diagnosis [41]. Another example is kidney transplant recipients who experienced a considerable decrease in N-specific IgG seroprevalence six months after infection [42]. Of interest, 60 people who participated in our study reported previous infection with SARS-CoV-2, but there was no evidence of seroconversion.

There is still considerable research work that should be done in this field [12]. First, it is important that the immune system and its response to SARS-CoV-2 infection, both in the acute phase and during convalescence, should be investigated in an integrated manner in longitudinal studies of large cohorts of asymptomatic, mild, moderate, and severe cases [28,39,43]. Circulating antibodies are one element of the adaptive immune response. Immune response and memory also consist of memory B cells, CD4 T cells, and CD8 T cells with their own kinetics [39]. Interrelationships of these immune components should be explored, in order to enhance our understanding with implications for protection from re-infections and for COVID-19 vaccines. The long-term persistence of seroconversion and immune memory in general, and the concentration and kinetics of neutralizing antibodies, which are important for sterilizing immunity, antibody levels that confer protection, innate immunity, and immune responses at local sites and portals of virus entry, are also domains that should be prioritized for future research [12,24,39,44].

Practical difficulties and critical challenges during the pandemic influenced the uniform representation of the population island-wide, with over-representation of the Nicosia prefecture, while there was a slightly higher percentage of females. In addition, our analyses depended on people’s reports of their past infection, which are subject to recall bias. However, our findings on seroconversion from the qualitative and quantitative antibody tests agree with the majority of the participants who reported a previous SARS-CoV-2 infection, while most individuals who did not report a previous infection were seronegative, as expected. Recall bias is also likely in our analyses because symptoms were not retrieved from medical records or surveillance registries but were reported by the participants during their interview for this study. Another weakness of this study is that it focused on the adult population, which limits our ability to understand antibody response among adolescents and children in Cyprus.

Future studies should be performed in a more systematic approach, to evaluate antibody and cellular immune responses of the Cypriot population to COVID-19 vaccination, with and without previous SARS-CoV-2 infection. Overall, our study provides information on the antibody response to natural SARS-CoV-2 infection in the Cypriot population and suggests associations with factors such as age, gender and symptom severity, which could help to provide insights into antibody-mediated immune protection and to be evaluated with future data on SARS-CoV-2 vaccine-mediated antibody levels.

## 5. Conclusions

Our work examines for the first time the antibody response to natural SARS-CoV-2 infection in the Cypriot population using both qualitative and quantitative antibody measurement methods. We demonstrate that IgG levels increase in the first three months post infection and then decrease but remain detectable more than six months post infection. Circulating IgG levels show substantial variability, partly explained by differences across convalescent individuals in terms of gender, age, development of symptomatic disease, and necessity for hospital care. Overall, our work provides information on the immunological response to SARS-CoV-2 infection that could help inform public health measures and interventions in Cyprus.

## Figures and Tables

**Table 1:** Characteristics of participants tested for immunoglobulin class G (IgG) antibodies against SARS-CoV-2 (November 2020–September 2021).

All (*n* = 1898)		Reported Past SARS-CoV-2 Infection *n* (%) 1112 (58.6%)	No History of SARS-CoV-2 Infection *n*(%) 786 (41.4%)	*p*-Value
**Age in years**				
Median (IQR)	46.0 (35.0–57.0)	45.0 (33.0–55.0)	47.0 (37.0–58.0)	<0.001 ‡
**Gender,** ***n* (%)**				
Female	770 (40.6)	670 (60.2)	456 (58.0)	0.607 ¥
Male	1126 (59.3)	441 (39.7)	329 (41.9)	
Not Reported	2 (0.1)	1 (0.1)	1 (0.1)	
**Place of residence,** ***n* (%)**				
Abroad	78 (4.1)	35 (3.2)	43 (5.5)	
Ammochostos	55 (2.9)	31 (2.8)	24 (3.1)	
Nicosia	1413 (74.5)	800 (71.9)	613 (77.9)	**<0.001 ¥**
Larnaca	140 (7.4)	79 (7.1)	61 (7.8)	
Limassol	150 (7.9)	125 (11.2)	25 (3.2)	
Paphos	61 (3.1)	41 (3.7)	20 (2.5)	
Not Reported	1 (0.1)	1 (0.1)	0 (0.0)	
**Nationality,** ***n* (%)**				
Cypriot	1789 (94.3)	1050 (94.4)	739 (94.0)	0.686 ¥
Other	104 (5.5)	60 (5.4)	44 (5.6)	
Not Reported	5 (0.2)	2 (0.2)	3 (0.4)	
**HCW,** ***n* (%)**				
Yes	314 (16.5)	123 (11.1)	191 (24.3)	**<0.001 ¥**
No	1584 (83.5)	989 (88.9)	595 (75.7)	
**Smoking,** ***n* (%)**				
Yes	317 (16.7)	158 (14.2)	159 (20.2)	
No	1571 (82.8)	944 (84.9)	627 (79.8)	**<0.001 ¥**
No Answer	10 (0.5)	10 (0.9)	0 (0.0)	

HCW: Health-care workers; ‡ *p*-value of Mann–Whitney *U* test; ¥ *p*-value of chi-squared test.

**Table 2:** Results of tests for anti-SARS-CoV-2 immunoglobulin class G (IgG) targeting the nucleocapsid protein (qualitative method; SARS-CoV-2 IgG—Abbott Laboratories) and the receptor binding domain of the spike protein (quantitative method; SARS-CoV-2 IgG II Quant—Abbott Laboratories).

All (*n* = 1898)		Reported Past SARS-CoV-2 Infection *n* (%) 1112 (58.6%)	Without Reported History of SARS-CoV-2 Infection *n* (%) 786 (41.4)	*p*-Value
**Vaccination against COVID-19-1 dose,** ***n* (%)**				
Yes 761 (40.1)		143 (12.9)	618 (78.6)	**<0.001 ¥**
No 1137 (59.1)		969 (87.1)	168 (21.4)	
**Vaccination against COVID-19-2 doses,** ***n* (%)**				
Yes 687 (36.2)		87 (7.8)	600 (76.3)	**<0.001 ¥**
No 1211 (63.8)		1025 (92.2)	186 (23.7)	
IgG test-Qualitative (S/C index)-*n* = 734 *		***n* = 622**	***n* = 112**	
≥1.4	436 (59.4)	431 (69.3)	5 (4.5)	**<0.001 ¥**
<1.4	298 (40.6)	191 (30.7)	107 (95.5)	
IgG test-Qualitative (S/C index)-*n* = 734 *		***n* = 622**	***n* = 112**	
Median (IQR)	2.2 (0.5–4.8)	2.9 (1.1–5.2)	0.03 (0.02–0.07)	**<0.001** ‡
IgG test Quantitative (AU/mL)-*n* = 1132 *		***n* = 969**	***n* = 163**	
≥50	930 (82.2)	909 (93.8)	21 (12.9)	**<0.001 ¥**
<50	202 (17.8)	60 (6.2)	142 (87.1)	
IgG test Quantitative (AU/mL)-*n* = 1132 *		***n* = 969**	***n* = 163**	
Median (IQR)	340.9 (105.2–994.2)	432.1 (182.4–1147.3)	0.0 (0.0–0.9)	**<0.001** ‡

S/C: Signal/cut-off; AU: Arbitrary units; * Results are shown for participants without vaccination against COVID-19; ‡ *p*-value of Mann-Whiney *U* test; ¥ *p*-value of chi-squared test.

**Table 3:** Association of participants’ attributes with the likelihood of a positive result in the qualitative assay for the detection of immunoglobulin class G (IgG) against the nucleocapsid protein of SARS-CoV-2 (SARS-CoV-2 IgG-Abbott Laboratories) and with levels of receptor binding domain (RBD)-specific IgG antibodies (quantitative method; SARS-CoV-2 IgG II Quant-Abbott Laboratories). The analysis involved unvaccinated people with a self-reported history of laboratory-confirmed SARS-CoV-2 infection.

	IgG Test–Qualitative (S/C Index) (No History of Vaccination against COVID-19)				IgG Test–Quantitative (AU/mL) (No History of Vaccination against COVID-19)	
	*n* = 622				*n* = 969	
	≥1.4	<1.4	*p* Value ¥	OR (95% CI)	Median (IQR)	*p* Value ‡
**Age**						
0–19	10 (2.3)	17 (8.7)		Ref	425.4 (205.8–788.2)	
20–59	317 (73.6)	159 (83.3)		**3.4 (1.5–7.6)**	353.3 (157.8–869.2)	
≥60	104 (24.1)	15 (7.8)	**<0.001**	**11.8 (4.6–30.5)**	1413.7 (567.4–3267.5)	**<0.001**
**Gender**						
Female	242 (56.2)	125 (65.5)		Ref	368.3 (166.0–909.7)	
Male	189 (43.9)	66 (34.5)	**0.030**	**1.5 (1.1–2.1)**	529.8 (206.8–1655.9)	**<0.001**
**Symptoms**						
Yes	417 (96.8)	170 (89.0)		**3.7 (1.8–7.4)**	441.8 (187.1–1165.6)	
No	14 (3.3)	21 (11.0)	**<0.001**	Ref	264.2 (46.9–636.5)	**0.001**
Fever						
Yes	160 (37.1)	51 (26.7)		**1.6 (1.1–2.4)**	577.5 (216.7–1866.8)	
No	271 (62.9)	140 (73.3)	**0.011**	Ref	374.5 (161.0–970.8)	**<0.001**
Cough						
Yes	224 (52.0)	66		**2.0 (1.4–2.9)**	578.2 (223.3–1591.0)	
		(34.6)				
No	207 (48.0)	125 (65.5)	**<0.001**	Ref	349.9 (153.6–829.8)	**<0.001**
Shortness of breath						
Yes	121 (28.1)	45 (23.6)		1.3 (0.9–1.9)	621.1 (216.7–1912.3)	
No	310 (71.9)	146 (76.4)	0.240	Ref	383.2 (166.0–966.7)	**<0.001**
Muscle aches						
Yes	228 (52.9)	89 (46.6)		1.3 (0.9–1.8)	418.8 (189.6–1164.9)	
No	203 (47.1)	102 (53.4)	0.147	Ref	443.4 (167.6–1122.0)	0.862
Sore throat						
Yes	133 (30.9)	52 (27.2)		1.2 (0.8–1.7)	397.9 (186.5–1148.9)	
No	298 (69.1)	139 (72.8)	0.361	Ref	442.9 (178.4–1145.7)	0.694
Loss of taste						
Yes	222 (51.5)	89 (46.6)		1.2 (0.9–1.7)	415.8 (184.7–1108.1)	
No	209 (48.5)	102 (53.4)	0.258	Ref	440.9 (175.4–1205.1)	0.816
Loss of smell						
Yes	244 (56.6)	96 (50.3)		1.3 (0.9–1.8)	381.9 (170.7–1064.8)	
No	187 (43.4)	95 (49.7)	0.142	Ref	471.0 (194.8–1321.9)	0.135
Headache						
Yes	237 (55.0)	97 (50.8)		1.2 (0.8–1.7)	427.1 (180.7–1144.6)	
No	194 (45.0)	94 (49.2)	0.332	Ref	439.7 (182.4–1204.7)	0.877
Fatigue						
Yes	284 (65.9)	115 (60.2)		1.3 (0.9–1.8)	438.1 (186.5–1246.7)	
No	147 (34.1)	76 (39.8)	0.173	Ref	406.7 (170.4–1036.6)	0.100
Red feet toes						
Yes	3 (0.7)	1 (0.5)		1.3 (0.1–12.9)	415.0 (236.0–814.4)	
No	428 (99.3)	190 (99.5)	0.639	Ref	432.1 (180.7–1148.9)	0.947
Nausea and Vomiting						
Yes	64 (14.9)	17 (8.9)		**1.8 (1.2–3.1)**	460.9 (212.4–1538.8)	
No	367 (85.1)	174 (91.1)	**0.042**	**Ref**	425.4 (173.8–1108.1)	0.109
Diarrhea						
Yes	95 (22.0)	35 (18.3)		1.3 (0.8–1.9)	666.8 (270.0–1602.2)	
No	336 (78.0)	156 (81.7)	0.293	Ref	384.7 (163.1–1038.3)	**<0.001**
**More than two Symptoms**						
Yes	389 (89.4)	152 (79.6)		**2.4 (1.5–3.8)**	443.4 (187.3–12,608.5)	
No	42 (10.6)	39 (20.4)	**<0.001**	Ref	354.1 (115.1–758.0)	**<0.001**
**HCW**						
Yes	52 (12.1)	30 (15.7)		0.7 (0.5–1.2)	331.8 (136.7–850.9)	
No	379 (87.9)	161 (84.3)	0.216	Ref	441.8 (186.5–1166.7)	**0.048**
**Hospital Admission**						
Yes	34 (7.9)	6 (3.1)		**2.6 (1.1–6.4)**	2832.2 (1325.5–4165.4)	
No	397 (92.1)	185 (96.9)	**0.026**	Ref	388.1 (171.8–989.0)	**<0.001**
**Difference between dates of reported 1st SARS-CoV-2 diagnostic test and IgG test**						
≤1 month	29 (6.7)	7 (3.7)		**4.5 (1.6–12.5)**	447.9 (194.2–1318.2)	
(1–3) months	312 (72.6)	97 (50.8)		**3.5 (1.9–6.6)**	488.5 (216.2–1365.5)	
(3–6) months	68 (15.8)	64 (33.5)		1.2 (0.6–2.3)	341.6 (149.4–856.2)	
>6 months	21 (4.9)	23 (12.0)	**<0.001**	Ref	327.3 (163.6–874.8)	**<0.001**
**Difference between dates of symptom onset and IgG test**						
≤1 month	30 (7.0)	7 (3.7)		**5.5 (2.1–14.3)**	447.5 (207.6–1363.9)	
(1–3) months	303 (70.5)	89 (46.6)		**4.4 (2.6–7.4)**	495.0 (222.6–1407.0)	
(3–6) months	66 (15.3)	55 (28.8)		1.5 (0.9–2.8)	354.0 (153.6–894.4)	
>6 months	31 (7.2)	40 (20.9)	**<0.001**	Ref	300.8 (120.8–890.0)	**<0.001**

S/C: signal/cut-off; AU: arbitrary units; HCW: health-care workers; ¥ *p*-value of chi-squared or fisher exact test; ‡ *p*-value of Kruskal–Wallis test or Mann–Whitney *U* test.

**Table 4:** Multivariable analyses of levels of anti-SARS-CoV-2 receptor binding domain-specific immunoglobulin class G (IgG) antibodies (logarithm base 10-transformed) against various factors and covariates. The analysis involved 909 unvaccinated individuals with a reported history of SARS-CoV-2 infection.

	(1)	(2)	(3)	(4)
Variables	Model 1– Characteristics of Participants	Model 2–Symptoms	Model 3–Hospital Admission	Model 4–Overall
				
**Fever (Yes)**		0.149 ***		0.100 **
		(0.044)		(0.042)
**Cough (Yes)**		0.143 ***		0.109 ***
		(0.043)		(0.040)
**Shortness of breath (Yes)**		0.112 **		0.028
		(0.048)		(0.046)
**Diarrhea (Yes)**		0.132 ***		0.075
		(0.050)		(0.047)
**Symptoms Duration (in days)**		0.002 **		0.001
		(0.001)		(0.001)
**At Least two Symptoms (Yes)**		−0.029		0.116 *
		(0.081)		(0.068)
**Time difference between date of symptoms and date of antibody test**		−0.0011 ***		
		(0.000)		
**Age (in Years)**	0.011 ***			0.020 ***
	(0.001)			(0.001)
**Gender (Female)**	−0.134 ***			−0.102 **
	(0.040)			(0.040)
**BMI (kg/m^2^)**	0.019 ***			0.015 ***
	(0.004)			(0.004)
**Smoking Status (Yes)**	−0.238 ***			−0.201 ***
	(0.054)			(0.055)
**Health Care Workers (Yes)**	−0.107 *			−0.064
	(0.062)			(0.061)
**Time difference between date of first diagnostic test for SARS-CoV-2 (antigen-based or molecular) and date of antibody test**	−0.001 ***			−0.001 ***
	(0.000)			(0.000)
**Hospital Admission (Yes)**			0.734 ***	0.415 ***
			(0.080)	(0.082)
**Constant**	1.891 ***	2.591 ***	2.613 ***	1.793 ***
	(0.112)	(0.081)	(0.020)	(0.125)
				
**Observations**	930	833	956	844
**R-squared**	0.170	0.080	0.081	0.251

Standard errors in parentheses *** *p* < 0.001, ** *p* < 0.05, * *p* < 0.1.

## Data Availability

Data have not been deposited in a freely accessible database. Data and biological material are available on request by the University of Cyprus Biobank, at biobank@ucy.ac.cy.
